# Pooled analysis of T2 Candida for rapid diagnosis of candidiasis

**DOI:** 10.1186/s12879-019-4419-z

**Published:** 2019-09-11

**Authors:** Dong-Lan Tang, Xiao Chen, Chang-Guo Zhu, Zhong-wei Li, Yong Xia, Xu-Guang Guo

**Affiliations:** 1 Department of Clinical Medicine, The Third Clinical School of Guangzhou Medical University, Guangzhou, Guangdong China; 2 Department of Clinical Laboratory Medicine, The Third Affiliated Hospital of Guangzhou Medical University, Guangzhou, Guangdong China; 3 Key Laboratory for Major Obstetric Diseases of Guangdong Province, Guangzhou, 510150 China; 4 Key Laboratory of Reproduction and Genetics of Guangdong Higher Education Institutes, Guangzhou, 510150 China

**Keywords:** T2 Candida, Candida infection, Diagnosis, Meta-analysis

## Abstract

**Background:**

The present meta-analysis examined the diagnostic accuracy of T2 Candida for candidiasis.

**Methods:**

The literature databases, such as PubMed, Embase, DVIO, Cochrane library, Web of Science, and CNKI, were searched on T2 Candida detection.

**Results:**

A total of 8 articles, comprising of 2717 research subjects, were included in the study. The pooled sensitivity and specificity were 0.91 (95% confidence interval (CI): 0.88–0.94) and 0.94 95% CI: 0.93–0.95), respectively. The pooled positive likelihood ratio and negative likelihood ratio was 10.16 (95% CI: 2.75–37.50) and 0.08 (95% CI: 0.02–0.35), respectively. The combined diagnostic odds ratio is 133.65 95% CI: 17.21–1037.73), and the AUC of SROC is 0.9702 [(SE = 0.0235), Q* = 0.9201(SE = 0.0381)].

**Conclusions:**

The current evidence supported that T2 Candida has high accuracy and sensitivity and is of major clinical significance in the diagnosis of Candida infection.

## Background

Candidiasis is a major medical problem in the twenty-first century. The hospital-acquired candidiasis (candidemia and other forms of invasive candidiasis) is a life-threatening disease in patients with low immune function and severe illness [1, 2]. Presently, it is the fourth leading cause of nosocomial infections in the USA. Patients with candidiasis have about 50% mortality, and each incident would cause an extra healing cost of >$40,000 [3, 4]. In summary, despite the use of several antifungal agents, candidiasis is generally considered as a disease associated with medical advances and a leading cause of morbidity and mortality in the healthcare environment. This phenomenon might be attributed to the difficulty in diagnosing candidiasis and challenges in administering earlier and adequate antifungal therapy [5].

The delay in diagnosis and treatment of candidiasis is closely linked to the increase in mortality and healthcare costs, and hence, a rapid and effective detection method is an urgent requirement. Therefore, finding a rapid and sensitive diagnostic method that can directly test the entire clinical sample is essential. The technology known as T2 Candida investigated in the present study is expected to solve this problem.

The T2Candida Panel (T2 Biosystems) is an FDA-approved rapid diagnostic method that can detect candida pathogens in whole blood specifically and sensitively without the need for culture or nucleic acid extraction steps [6]. It uses T2MR technology [7]. The detecting density of T2MR is 1 colony forming unit (CFU)/mL of whole blood [6, 8]. Herein, we aimed to evaluate the diagnostic accuracy of T2 Candida by conducting a meta-analysis of the data extracted from relevant studies.

## Methods

### Database and literature search strategies

We searched the PubMed, Embase, OVID, Cochrane library, Web of Science, and China National Knowledge Infrastructure (CNKI) databases up to February 2018 and the official website of T2 Biosystems which developed T2 Candida, using the search phrase (T2 Magnetic Resonance OR T2MR OR T2 Candida OR T2Dx) AND (Candidiasis OR Candida OR Candidemia OR candidaemia OR fungemia). In addition, we scanned the references of all the studies included. The search results show that the latest research was updated in 2018 and the publication years ranged from 2013 to 2018.

### Inclusion/exclusion conditions

In order to assess the diagnostic accuracy of T2 Candida, we conducted prospective or retrospective cohort and case-control studies and included those reporting true-positives (TP), false-positives (FP), false-negatives (FN), and true-negatives (TN) or the studies where these variables could be calculated from the other published data.

The studies that were duplicated publications or have no description of the available data and had no restrictions on the language, publication status, year of study, or participants’ ages were excluded. All records were independently selected by authors according to the inclusion criteria, and a consensus was found on each record.

### Data extraction

Based on the inclusion conditions, two reviewers extracted the data from the studies separately. A third reviewer resolved the disagreements by discussion and in consultation.

### Quality assessment

The risk of bias was assessed independently by two reviewers using the Review Manager 5.2 (Copenhagen: The Nordic Cochrane Centre, The Cochrane Collaboration, 2012) software according to the Quality Assessment of Diagnostic Accuracy Studies 2 (QUADAS-2). There were four parts: patient selection, index test, reference standard, and flow and timing [9]. Each part was assessed in terms of the risk of bias; the first three parts were also rated based on the concerns about applicability. According to the “yes,” “no,” or “uncertain” responses of the relevant landmark questions included in each part, the “risk,” “high,” or “uncertainty” might be determined corresponding to the risk level of bias. The applicability sections are based primarily on the degree of matching with the appraisal problem. Concerns about the applicability are rated as “low,” “high,” or “unclear.” All qualified publications are independently rated by two review authors for quality. The disagreements were resolved by discussion and in consultation with a third reviewer.

### Statistical analysis

Statistical analyses were performed using The Meta-disc software, version 1.4 [10]. We extracted the TP, FP, TN, and FN from each study, plotted the summary receiver operating characteristic (SROC) curve, and estimated the absolute diagnostic accuracy of each test. Spearman’s correlation coefficient was assessed to evaluate the heterogeneity of the included studies (threshold effects and non-threshold effects). The random-effect model was used if significant heterogeneity existed among the individual studies. In addition, the fixed-effect model was used to calculate the pooled sensitivity, specificity, positive likelihood ratio, negative likelihood ratio, and the diagnostic odds ratio of the included studies. To assess the publication bias of the included studies, we used STATA 14.0 (StataCorp, College Station, Texas, USA) to perform Deeks’ regression test of funnel plot asymmetry and the Egger’s test.

## Results

### Summary of the included studies

**Fig. 1:**
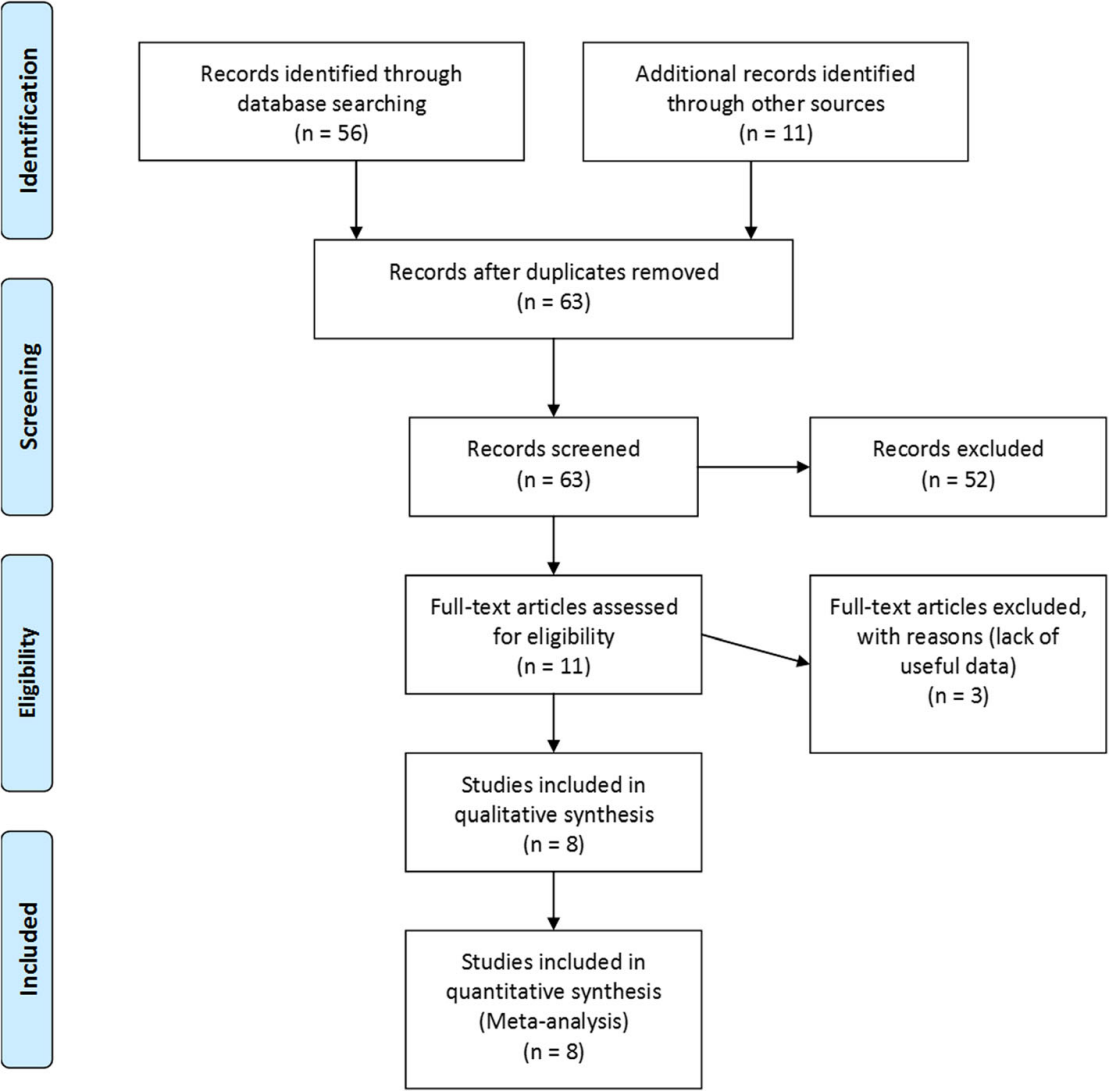
Flow Chart of the Study Selection Process

The following literature databases were searched: PubMed, Embase, OVID, Cochrane library, Web of Science, CNKI, and T2 Biosystems web pages, depending on the keywords. A total of 63 related documents were discovered. After reviewing the abstract, 52 studies that did not involve the detection efficiency of Candida were removed. The remaining articles examined the literature and excluded 3 studies due to the lack of experimental sensitivity and specificity. Finally, a total of 8 articles consisting of 2717 research subjects were included [6–8, 11–15] (Fig. 1). The relevant data in each literature were extracted according to the proposed effect-indicators (the study of *C. albicans*, *C. tropicalis*, *C. parapsilosis*, *C. krusei*, and *C. glabrata*) (Table 1).

**Table 1:** Summary of the included studies

Diagnostic Test	Author /year	Method	Size	Sample type	Country	TP	FP	FN	TN
T2Candida	Neely 2013 [6]	Culture	133	Whole blood	America	88	0	2	43
T2Candida	Mylonakis 2015 [8]	Culture	1801	Whole blood	America	233	29	23	1516
T2Candida	Beyda 2013 [13]	Culture	270	Whole blood	America	90	4	0	176
T2Candida	Hamula 2016 [14]	Culture	24	Whole blood	Athens	15	0	0	9
T2Candida	Dwivedi 2016 [12]	Culture	200	Whole blood	America	11	22	17	150
T2Candida	Pappas 2015 [11]	Culture	91	Whole blood	America & England	3	14	0	74
T2Candida	Mylonakis 2018 [15]	Culture	46	Whole blood	America	7	16	0	23
T2Candida	Clancy 2018 [7]	Culture	152	Whole blood	America	32	37	4	79

### Quality of the included study

**Fig. 2:**
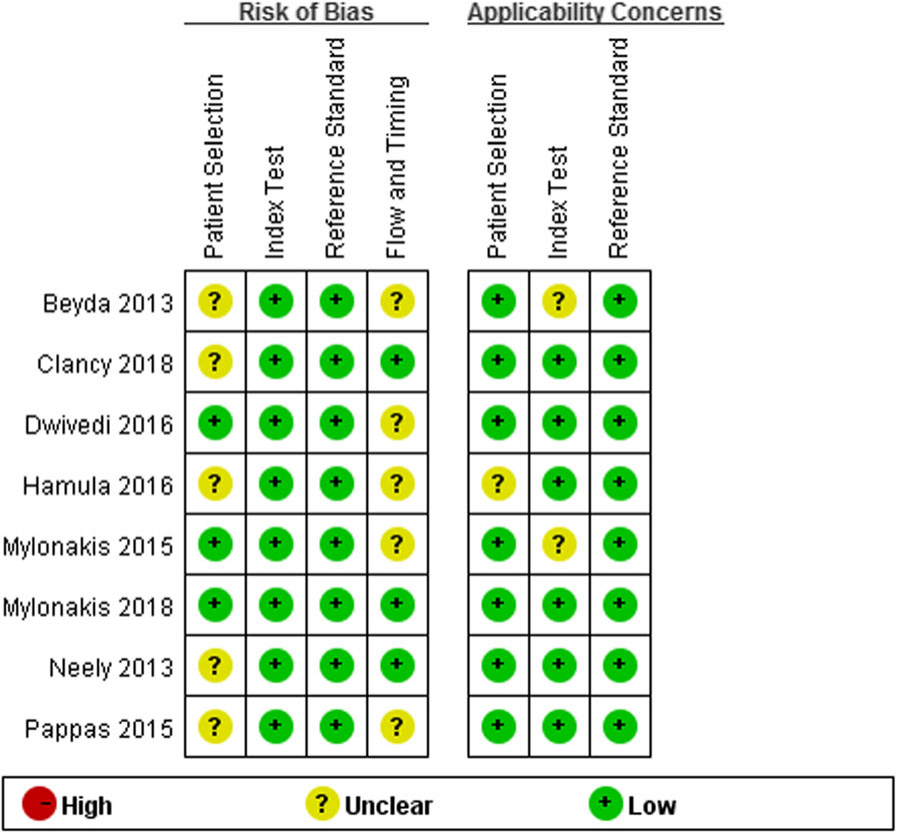
Quality evaluation plot of methodological

Being dependent on the results of the QUADAS-2 (Fig. 2), the risk of bias in the study was low, and the clinical applicability was low.

### Risk analysis of publication bias

**Fig. 3:**
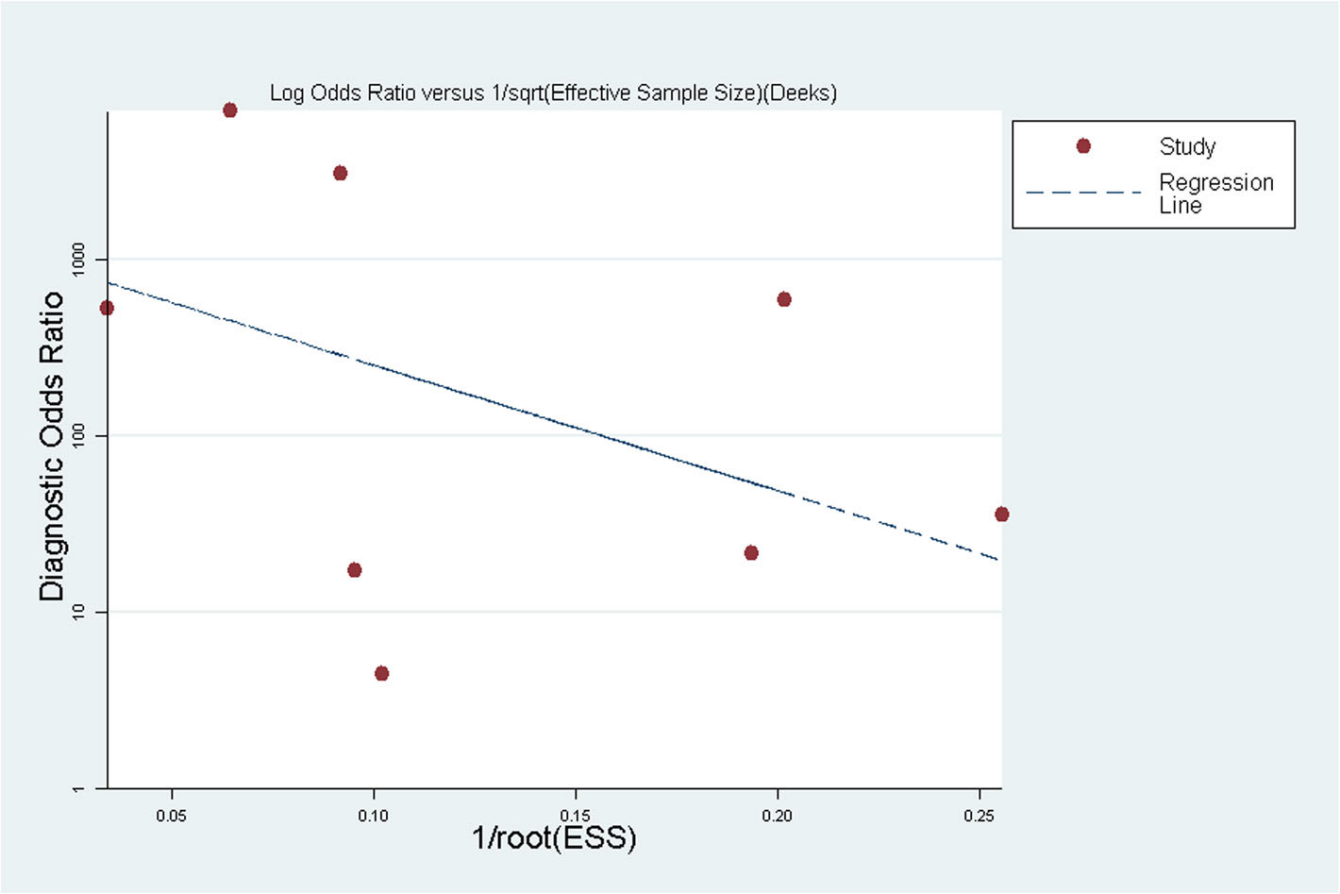
Publication bias Plot for T2Candida

No publication bias was discovered in the funnel plot (Fig. 3). The Egger’s test showed that the publication bias of the study was small (*P* = 0.404).

### Threshold effects

**Fig. 4:**
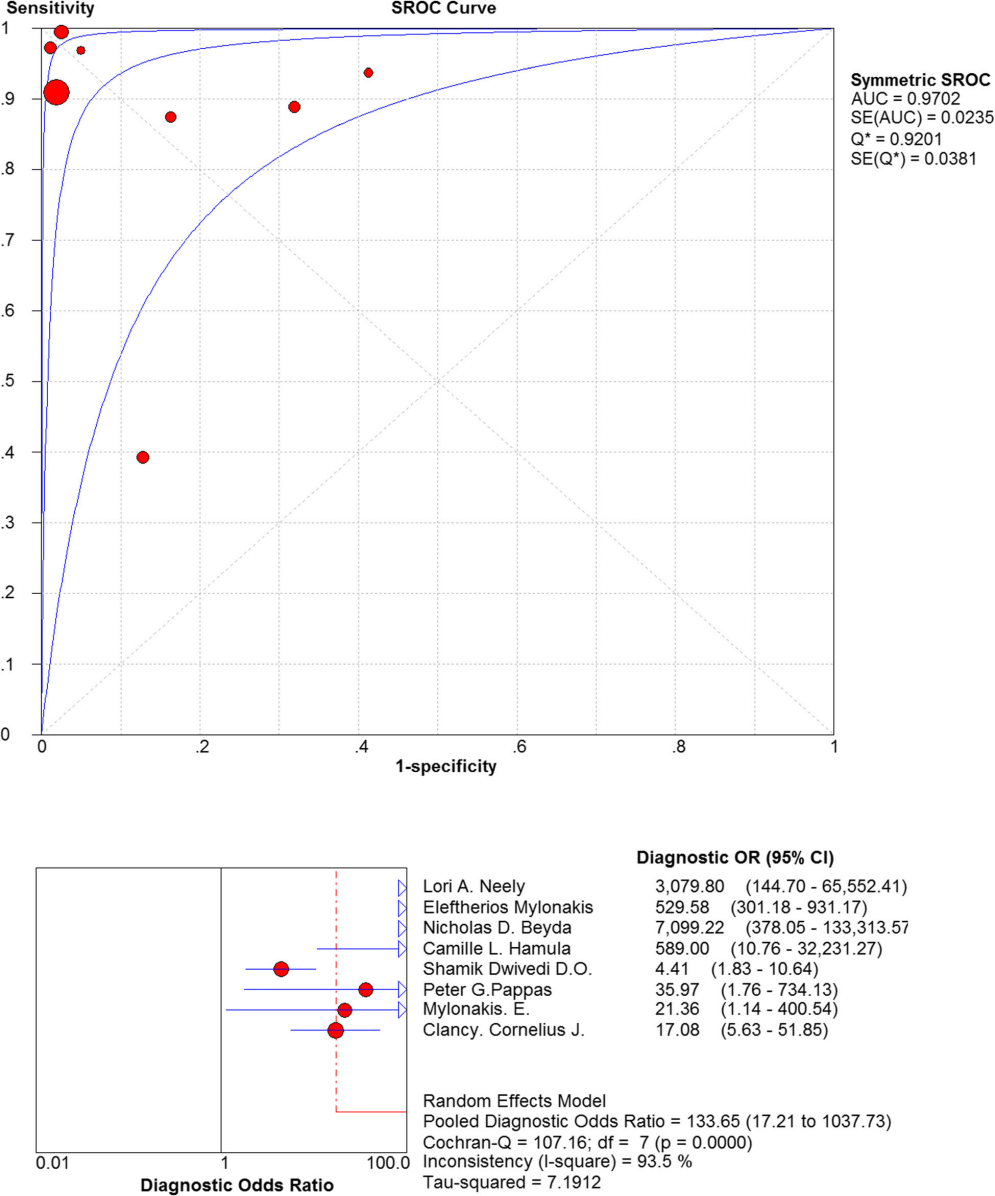
Summary receiver operating curves and forest plot of diagnostic odds ratio for T2Candida

Firstly, the SROC curves were examined, and the scatter plots did not appear as “shoulder-arms” in the image formed on the SROC curve, indicating the absence of threshold effect (Fig. 4). Secondly, the Spearman’s correlation coefficient of the logarithm of sensitivity and specificity was = − 0.500, *P*-value = 0.207, which also indicated that there was no threshold effect in the included studies. Consequently, there is no threshold effect stated among the articles constituted in the present analysis. In terms of thresholds, each of the included studies was homogeneous.

### Non-threshold effects

**Fig. 5:**
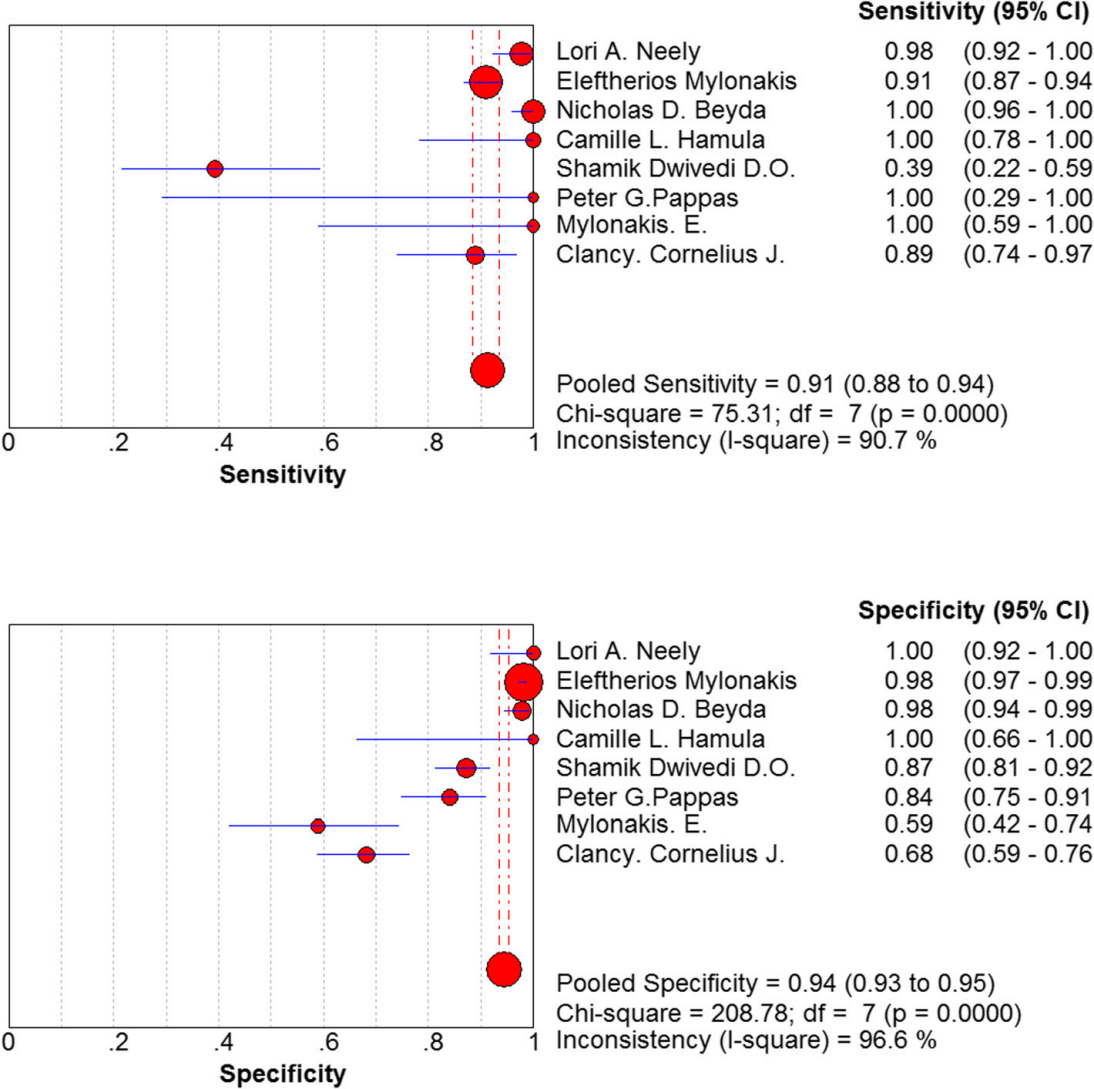
Forest plots of sensitivity and specificity for T2Candida

The non-threshold effect test was assessed using the χ^2^ test. The heterogeneity caused by non-threshold effects was evaluated using a diagnostic odds ratio (DOR). Cochran-Q = 107.16, *P* < 0.05 indicated a non-threshold effect heterogeneity among the included studies (Fig. 4). Another analysis indicated that the combined sensitivity was: χ^2^ = 75.31, I^2^ = 90.7%, the combined specificity was: χ^2^ = 208.78, I^2^ = 96.6%. These values confirmed the non-threshold effect of heterogeneity between the included studies (Fig. 5).

### Diagnostic accuracy of T2 Candida for candidiasis

**Fig. 6:**
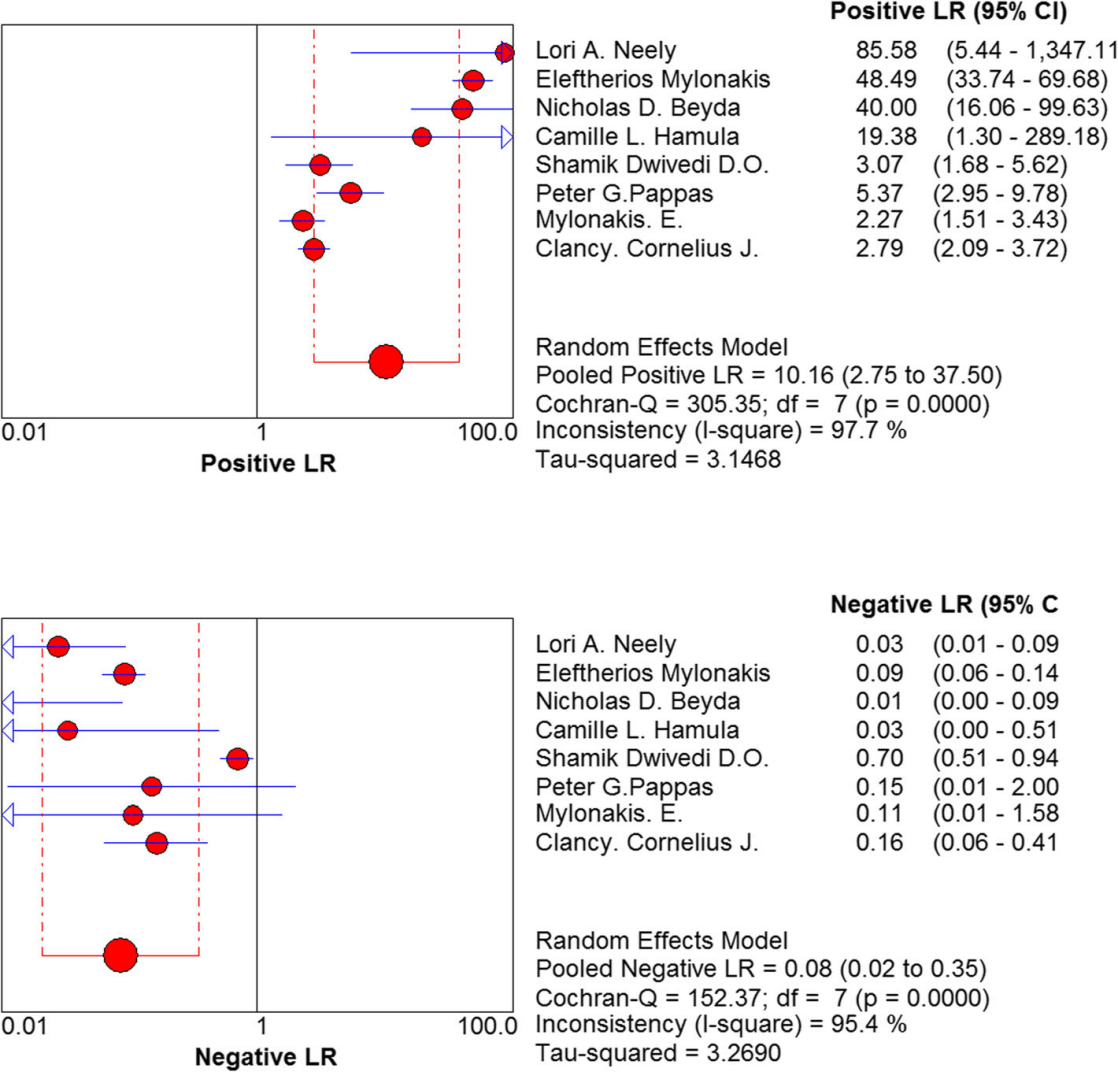
Forest plots of positive likelihood ratio and negative likelihood ratio for T2Candida

The pooled diagnostic ratio was 133.65 (95% confidence interval (CI): 17.21–1037.73) (Fig. 4), and the area under the SROC curve was: AUC = 0.9702 (SE = 0.0235), Q* = 0.9201 (SE = 0.0381). The pooled sensitivity of T2 Candida in the differential diagnosis of candidiasis was 0.91 (95% CI: 0.88–0.94), and the pooled specificity was 0.94 (95% CI: 0.93–0.95) (Fig. 5). The pooled positive likelihood ratio was as follows: 10.16 (95% CI: 2.75–37.50). The pooled negative likelihood ratio was as follows: 0.08 95% CI: 0.02–0.35) (Fig. 6).

## Discussion

Blood culture has been considered as the diagnostic gold standard for invasive candidiasis. Nonculture diagnostic tests, such as an antigen, antibody, or β-D-glucan detection assays and polymerase chain reactions (PCR) are applied as the supplements to cultures in clinical practice.

The overall sensitivity of blood cultures for diagnosing invasive candidiasis is about 50% [16]. Blood cultures have a limitation; the slow turnaround time (median time to positivity of 2–3 days, ranging from 1 to ≥7 days) leads to a delay in diagnosis [17, 18]. Currently, *Candida* antigen has been detected by the anti-*Candida* antibody. The combined mannose/anti-mannose antibody assay is the optimal method using *Candida* antigen and anti-*Candida* antibody detection. The sensitivity/specificity of diagnosing invasive mannose candidiasis was 58%/93%, and the rate of combined determination was 83% according to a meta-analysis conducted on 14 studies [19]. In another technology, the anti-human IgG detection was 59%/83 and 86%, respectively [20]. However, antigen detection has a limitation as they can easily clear from the bloodstream [21]. The reliability of antibody detection in immunosuppressive hosts is poor, and hence, this assay is rarely used in the USA. The sensitivity and specificity for the diagnosis of invasive candidiasis were 75–80 and 80%, respectively based on a meta-analysis of β-D-glucan studies [22–24]. The true-positive results of β-D-glucan detection are not specific for invasive candidiasis, thereby indicating the possibility of an invasive fungal infection. Thus, the detection of β-D-glucan has poor specificity and sensitivity. In a recent meta-analysis, the sensitivity and specificity of PCR for suspecting invasive candidiasis were 95 and 92%, respectively [25], and among the putative invasive candidiasis, the sensitivities of PCR and blood culture were 85 and 38%, respectively. A major limitation of PCR studies is the lack of standardized methodologies and multicenter validation of the assay [5].

*Candida sp.* is a conditional pathogen that infects the body and can cause various diseases. It is a common infection in hospitals. According to the study from the ARTEMIS DISK Global Antifungal Surveillance Study, 1997–2007, > 90% of the invasive diseases, such as candidiasis are triggered by the 5 most common pathogens: *C. albicans*, *C. tropicalis*, *C. parapsilosis*, *C. krusei*, and *C. glabrata*. [26]. In order to reduce the increased cost of treatment owing to the delay in diagnosis and reduce the poor prognosis of candidiasis caused by the delay in diagnosis and treatment, a rapid and accurate diagnosis is required urgently. Present, the emergence of new diagnostic technology, T2 Candida, can resolve this long-standing clinical problem. T2 Candida is the first FDA-approved rapid and automated molecular diagnostic test to detect Candida sp. directly from blood, being culture-free.

In the blood sample test, compared to the blood cultures (gold standard), T2 Candida can detect the equivalent proportion of *Candida*, and the test time decreased from an average of 2.6 days in blood culture to about 3–4 h [13]. Thus, this technology is significantly superior to the gold standard (BC) in the identification of candidiasis [27]. Compared to the other diagnostic tests, high combined sensitivity (91%) and specificity (94%) are noted. This can also lead to better antifungal stewardship (good thing to include) [28]. Nevertheless, according to the designer’s statement, T2 Candida can only detect 5 *Candida* species: *C. albicans*, *C. tropicalis*, *C. parapsilosis*, *C. krusei*, and *C. glabrata*. It’s the limitation of T2 Candida. But > 90% of the invasive diseases, such as candidiasis are triggered by these 5 pathogens [26]. So T2 Candida is a highly valued detecting tool.

By searching the relevant literature on technology and collecting the relevant data, we combined and analyzed the diagnostic test data of T2 Candida: pooled sensitivity: 0.91 (95% CI: 0.88–0.94), pooled specificity: 0.94 (95% CI: 0.93–0.95). Strikingly, the combined sensitivity and specificity are both high. Furthermore, the resulting SROC curve is distant from the middle diagonal and close to the upper left corner, and the AUC = 1.0, which indicates an improved accuracy of T2 Candida is better.

We were also concerned about the heterogeneity. This study has developed stringent criteria for the inclusion and exclusion of the studies, minimizing the sources of heterogeneity. I^2^ values of the pooled sensitivity and specificity were > 90%, indicating a large heterogeneity among the included studies. In terms of threshold effects, the included studies were homogenous as assessed by statistical analysis. However, in the non-threshold effect analysis, a non-threshold effect heterogeneity was detected between the included studies. Markedly, this meta-analysis could not perform subgroup analysis to explore its heterogeneity due to time constraint and the small number of studies. According to the included literature, heterogeneity caused by the non-threshold effects originated from the factors, such as disease severity and concomitant diseases and test conditions such as different technologies, tests, operators, standard tests, and age (adults or children). However, the sensitivity of T2 Candida obtained by the study of Shamik et al. was 39% [12], and the specificity of the study by Mylonakis et al. was 59% [8], which deviated significantly from the pooled prediction values. These characteristics were related to several factors, such as the performance of T2 Candida, predicting patients’ outcomes, and cost-efficiency in various settings.

In conclusion, T2 Candida, the novel detection technology, has high efficiency, high specificity and time efficiency.

## Conclusions

In summary, the current meta-analysis suggested that T2 Candida can be considered as a novel detection technology with high sensitivity and specificity. The method had a rapid and accurate diagnostic ability, a potential to improve the prognosis of the disease, reduce unnecessary expenses, and shorten the detection period. Thus, the T2 Candida could be a significant improvement for the laboratory diagnosis of candidiasis.

## Data Availability

All data generated or analysed during this study are included in this published article [and its supplementary information files].

## Abbreviations

AUC: Area under curve
BC: Blood culture
C: Candida
CI: Confidence interval
DOR: Diagnostic odds ratio
Fig: Figure
FN: False-negatives
FP: False-positives
ROC: Receiver operating characteristic
SE: Standard error
SROC: Summary receiver operating characteristic
TN: True-negatives
TP: True-positives
